# Study on the Properties of Carbon Nanotube (CNTs) Reinforced AlSi10Mg Composites Fabricated by Powder Metallurgy

**DOI:** 10.3390/ma16113905

**Published:** 2023-05-23

**Authors:** Jian Pan, Linchi Zou, Zengxiang Liao, Zhijie Lin, Junfeng Chen

**Affiliations:** 1 College of Materials Science and Engineering, Fujian University of Technology, 3 Xueyuan Road, University Town, Fuzhou 350118, China; pj18756863240@163.com (J.P.); zx2221604027@163.com (Z.L.); zjlin@fjut.edu.cn (Z.L.); 2 Fujian Provincial Key Laboratory of Advanced Materials Processing and Application, 3 Xueyuan Road, University Town, Fuzhou 350118, China; 3 School of Materials Science and Engineering, Qishan Campus, Fuzhou University, 2 Xueyuan Road, University Town, Fuzhou 350116, China

**Keywords:** CNT/AlSi10Mg composites, powder metallurgy, mechanical properties, corrosion

## Abstract

The objective of this study is to prepare CNT/AlSi10Mg composites using mechanical ball milling combined with SPS. The study investigates the influence of ball-milling time and CNT content on the mechanical and corrosion resistance of the composite. This is performed to address the challenge of CNTs dispersion and to understand how CNTs impact the mechanical and corrosion resistance of the composites. The morphology of the composites was characterized using scanning electron microscopy (SEM) transmission electron microscopy (TEM) and Raman spectroscopy, and the mechanics and corrosion resistance of the composite materials were tested. The results demonstrate that the uniform dispersion of CNTs can significantly enhance both the mechanical properties and corrosion resistance of the material. Specifically, when the ball-milling time was 8 h, CNTs were uniformly dispersed in the Al matrix. The CNT/AlSi10Mg composite shows the best interfacial bonding when the mass fraction of CNTs is 0.8 wt.%, with a tensile strength of −256 MPa. This is 69% higher than the original matrix material without the addition of CNTs. Moreover, the composite exhibited the best corrosion resistance.

## 1. Introduction

Aluminum and alloys are widely used in fields such as aerospace, transportation, and other industries that demand high strength and lightweight materials due to their low density, high specific tensile strength, good corrosion resistance, and high thermal and electrical conductivity [1,2,3]. Aluminum matrix composites are lightweight, high-strength, and advanced composites that synergize with aluminum and its alloys by incorporating high-performance reinforcements [4,5]. Carbon nanotubes have excellent mechanical properties such as a high Young’s modulus, high strength, high thermal conductivity, low density, good chemical stability, and a self-lubricating effect, making them promising reinforcing materials for metal matrix composites [6,7]. The main challenge in the preparation of CNT/Al matrix composites is to resolve the dispersion of CNTs and the interfacial bonding between CNTs and the Al matrix [8,9] The primary methods for preparing composite materials include in situ autogenous, molecular level mixing, stirring and friction, wet mixing, and powder metallurgy [8,10,11,12,13,14]. Among these methods, powder metallurgy is preferred by researchers due to its ability to disperse CNTs uniformly and maintain their integrity well. Commonly used powder metallurgy processes include cold pressure-less sintering, hot-pressure sintering, and discharge plasma sintering [3]. Kuzumaki et al. [15] used a hot-pressure sintering process to sinter solution-mixed Al-5 vol.% CNTs composite powder at 600 °C for 1.5 h. However, the poor dispersion of CNTs resulted in a tensile strength (TS) of only 80 MPa, which was even lower than that of pure aluminum. Nevertheless, the high-temperature stability of the prepared composites improved significantly compared to pure aluminum. Singh et al. [16] first ball-milled (180 r/min) aluminum powder for 10 h, sonicated (4 h) functionalized carbon nanotubes, mixed them with the powder, and then further ball-milled (100 r/min for 15 min). Then, Al–CNT (0.75 and 1.5 vol.%) composites were prepared by discharge plasma sintering. They achieved a TS of 217 MPa (161 MPa for Al) and a failure strain of 4.7% (6.6% for Al) for Al0.75 vol% CNT composites. Low ball-milling speed and low ball-milling time led to the presence of carbon nanoclusters, and the tensile properties of the Al-1.5 vol% CNT composites (126 MPa TS and 3.3% elongation) were poorer than those of the Al samples. Liu et al. [17] investigated the effect of different ball-milling times on the properties of CNT/Al composites, and it was found that mechanical ball milling from 8 to 12 h led to severe damage of the CNTs, resulting in interfacial reactions between CNT and aluminum to form Al_4_C_3_ and a decrease in the properties of the composites. The best mechanical properties of the composites were obtained when the ball-milling time was 6 h. Junhui Nie et al. [18] prepared composites with CNT content of 0–2 wt.% by mechanical ball milling mixing combined with discharge plasma sintering technique. The results show that the tensile strength and Vickers hardness of CNT/Al composites increase by 29.9% and 13.2%, respectively, when the CNT content was 0.5 wt.% compared to pure Al. The decrease in the mechanical properties of the CNT/Al composites with increasing CNT content was attributed to the presence of CNT clusters in the matrix. Run et al. [19] prepared CNT/Al composites with a CNT content of 1.5 wt.% by using a variable-speed ball-milling technique combined with sheet powder metallurgy. They found that the low speed combined with high-speed ball-milling method could better disperse CNTs on the surface of aluminum powder without causing serious damage to the structure of CNTs. The tensile strength of the composite increased from 311 MPa to 408 MPa for pure aluminum, and the elongation was reduced to 4%.

In summary, the use of mechanical ball milling combined with powder metallurgical methods can effectively disperse carbon nanotubes in the matrix to improve the mechanical properties of the material. However, most of the current studies focus on the dispersion of CNTs in the matrix, and the corrosion resistance of the composite is rarely reported. AlSi10Mg alloy is a widely used casting aluminum alloy with advantages such as good fluidity, low density, good casting properties, mechanical properties, and corrosion resistance, making it useful in various applications in aerospace and automotive industries [20,21,22]. Currently, composite materials with AlSi10Mg alloys as matrices are usually prepared by selective laser melting (SLM), and the mechanical properties of the composites prepared by this method are significantly improved. However, SLM is a type of liquid forming, and the reinforcing phase is destroyed at high temperatures and easily reacts with the matrix to produce the brittle phase Al_4_C_3_. Moreover, liquid forming is a type of casting process, and casting defects, such as porosity and shrinkage, are easily produced during the preparation process, so that the material is not dense enough [23]. For example, Wang et al. [24] prepared CNT/AlSi10Mg composites by mixing CNTs with AlSi10Mg and ball milling, followed by SLM, and it was found that the microhardness of the composites was 143.7 HV, and that the doping of carbon nanotubes improved the strength of Al matrix composites compared with unreinforced AlSi10Mg parts. However, the ductility decreased and the structure of CNTs was severely damaged. Jiang et al. [25] prepared 1.0 wt.% CNT/AlSi10Mg composites by adding CNTs to aluminum powder by ball milling and using the SLM technique, which increased the hardness by approximately 10% and tensile strength by approximately 20% owing to the presence of reinforced phase CNTs, but also generated the brittle Al_4_C_3_ phase during 3D printing.

In this work, AlSi10Mg was selected as the matrix alloy. The dispersion of CNTs by an efficient mechanical ball-milling process was performed, and the composites were prepared by a combination of spark plasma sintering (SPS). The ball-milling time and carbon nanotube content were investigated, and the microstructure, mechanical properties, and corrosion resistance of the composites were analyzed in-depth to provide a basis for the preparation of high-strength CNT/AlSi10Mg composites.

## 2. Materials and Methods

### 2.1. Preparation Methods

**Figure 1:**
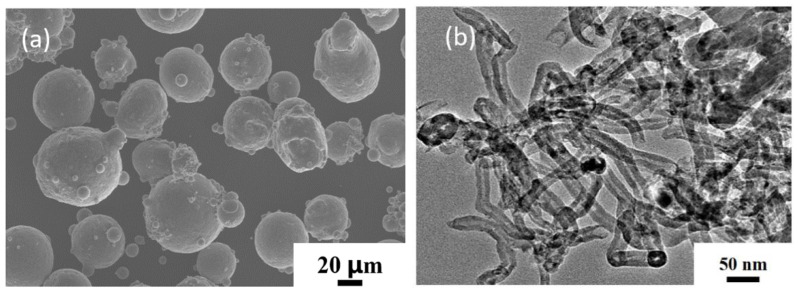
SEM images of (**a**) pristine aluminum powder. (**b**) Carbon nanotubes after acid washing.

**Figure 2:**
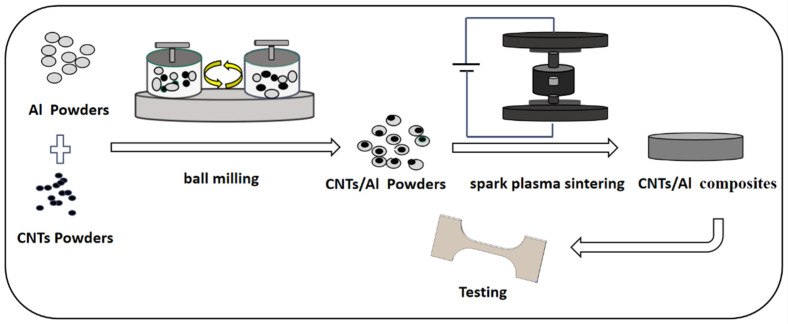
Schematic illustration for the processing route CNT/AlSi10Mg composite.

AlSi10Mg alloy spherical powder with an average diameter of 50 μm was used as the matrix material. Multi-walled CNTs with 97.5% purity, 10–20 nm diameter, and 5–15 μm length were used as the reinforcing material, which were provided by Shenzhen Nanotechnology Co. The morphologies of the raw materials are shown in Figure 1. The preparation process of the CNT/AlSi0Mg composites is illustrated in Figure 2. First, the CNTs were acid-washed in a H_2_SO_4_:HNO_3_ = 3:1 solution, and dried for 12 h. Subsequently, the AlSi10Mg and CNTs powders were ball-milled with stainless-steel balls in a stainless-steel tank using a planetary grinding machine at 220 rpm. The ball-to-powder weight ratio was 15:1, and the milling time was set to be 6, 7, 8, and 9 h. A process control agent (PCA) of anhydrous ethanol (20 mL) was added to prevent excessive cold welding of the powders. The composite powders were solidified in a spark-plasma fast-sintering furnace (SPS-5T-5-III, Shanghai Chenhua Technology Co., Ltd., Shanghai, China). The sintering temperature was controlled at 540 °C, the sintering pressure was 40 MPa, and the holding time was 18 min. Subsequently, the alloy samples were cooled to room temperature in a furnace to obtain uniform and dense CNT/AlSi10Mg composites. The samples were then gradually polished with 600# and 3000# sandpapers, cleaned with acetone, dried, and prepared for use.

### 2.2. Material Characterization

The microstructural characterization and phase composition of the composites, as well as the morphology of the tensile fractures, were analyzed using a Bruker D8 X-ray diffractometer equipped with an energy-dispersive spectrometer (XRD, Karlsruhe, Germany) and a field-emission scanning electron microscope (SEM, FEI-Nova Nano 450, Hillsboro, ON, USA) with an energy-dispersive spectrometer (EDS, Model Link-ISIS Oxford, UK). The microstructures of the composites were further analyzed using SEM and transmission electron microscopy (TEM, JEM-2100, 200 KV, manufacturer, Tokyo, Japan). The crystallographic structures of CNT/AlSi10Mg powders was tested using coaggregation Raman spectroscopy (DXR2xi, Thermo, OR, USA) with a laser wavelength of 514.5 nm. Tensile properties were measured using an electronic universal testing machine (Instron 2382, Engstrom, Boston, MA, USA) at a crosshead speed of 0.6 mm/min, with dogbone-shaped specimens having a length of 10 mm, width of 2 mm, and thickness of 1 mm. Three samples of each type of material were tested to obtain the yield strength (YS), ultimate tensile strength (UTS), and elongation. The corrosion resistance of the materials was tested using a three-electrode system and electrochemical workstation (CHI660E). A platinum electrode and a glycerol electrode (SCE, +0.2444 V) were used as the auxiliary and reference electrodes, respectively, with a 3.5 wt.% NaCl solution as the corrosion solution. The working surface of the electrochemical corrosion sample should be polished and then connected with copper wire on the reverse side and encapsulated so that only the working surface of the sample can contact the corrosion solution. The samples were then energized and tested to ensure good contact. After the experiment, the samples were cleaned in alcohol and dried for subsequent morphological observations.

## 3. Results and Discussion

### 3.1. Effects of Different Ball-Grinding Times on Composite Powders

#### 3.1.1. Morphology of CNT/AlSi10Mg Composite Powders at Various Ball-Milling Times

**Figure 3:**
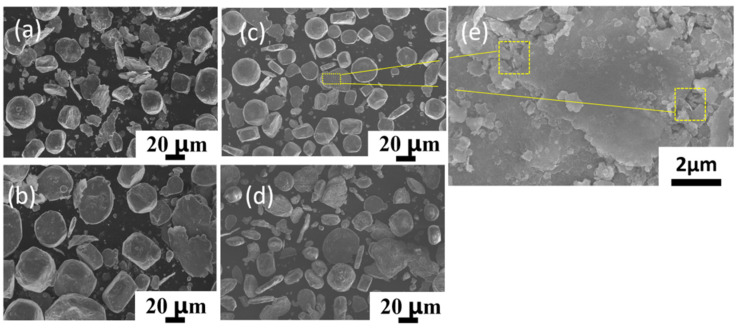
SEM images of 0.2% CNT/AlSi10Mg composite powder with different ball-milling times: (**a**) 6 h. (**b**) 7 h. (**c**) 8 h. (**d**) 9 h. (**e**) 8 h CNTs distribution.

**Figure 4:**
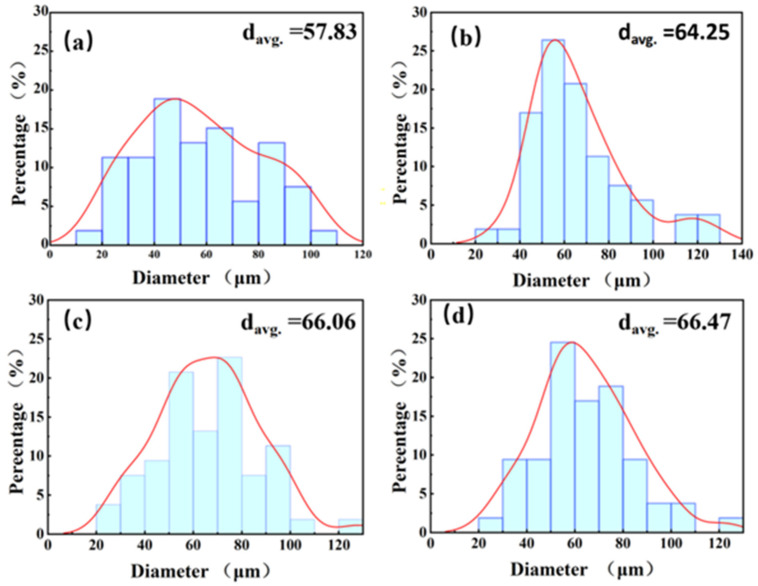
Particle size distribution of CNT/AlSi10Mg composite powder (**a**) 6 h. (**b**) 7 h. (**c**) 8 h. (**d**) 9 h.

Figure 3 shows the powder morphology at different ball-milling times. To increase the attachment surface for the CNTs, ball milling was performed at a low speed for a long time. This has the advantage that after a significant amount of time, the powder gradually becomes flaky, thereby increasing the surface area, while, at the same time, not significantly damaging the structure of the CNTs. As shown in the Figure, with the increase in ball-milling time from 6 h to 9 h, the powder gradually changes from spherical to flaky. Figure 3a shows that a large number of small particles gather together during the ball-milling process due to shear forces, becoming irregularly shaped, and the originally spherical powder became ellipsoidal. Figure 3b–d are the powder morphologies of 7 h, 8 h, and 9 h ball milling, respectively. With the increase in time, most of the ellipsoid powder is deformed into flat sheets, but the powder surface of 9 h ball milling becomes rougher, because anhydrous ethanol is added as the process control agent during powder mixing to prevent cold welding. Therefore, after a long time of ball milling, the surface of aluminum powder is oxidized to Al_3_O_2_ [26]. which makes the surface of the powder change. Compared with the ball milling for 6 h, the shape of the powder becomes more regular at other times, which is due to the fact that with the increase in ball-milling time, some small-sized powders agglomerate and are continuously squeezed into regular flakes. However, the ball-milling time should still be controlled within a reasonable range to prevent the heavy oxidation of the powder. Figure 3e shows the magnified morphology of ball milling for 8 h, in which CNTs can be clearly seen to be uniformly extruded in the Al powder, indicating that ball milling for 8 h can uniformly disperse CNTs. Figure 4 shows the graph of powder particle size at different times of ball milling. It can be observed that with the increase in ball-milling time, the powder particle size gradually becomes larger. This conclusion can also be proved by combining Figure 3, the average particle size of the original spherical powder, which is 50 μm, with the increase in milling time, the powder is constantly squeezed by collision and gradually becomes a flake, so the powder size also increases.

#### 3.1.2. Characterization of CNTs Integrity at Different Ball-Milling Times

**Figure 5:**
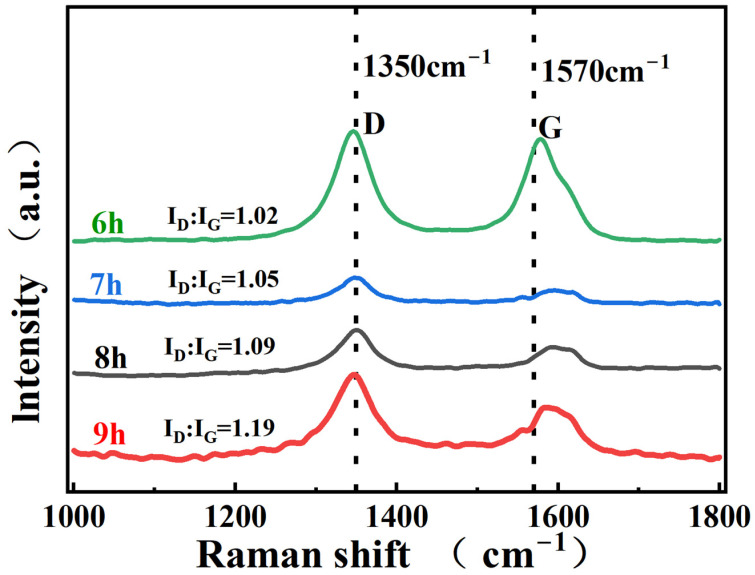
Raman spectra of CNT/AlSi10Mg composite powder with different ball-milling times.

To assess the structural integrity of the CNTs, Raman spectroscopy was used to test the composite powders. The representative peaks of CNTs are mainly the G peak at 1570 cm^−1^ and the D front at 1350 cm^−1^. The D-peak indicates the defective nature of the carbonaceous structure, and the G-peak indicates the integrity of the CNTs. Therefore, the I_D_/I_G_ ratio is commonly used to reflect the integrity of the CNTs [27]. The original I_D_/I_G_ of the acid-washed CNTs is 0.98, and Figure 5 shows that the I_D_/I_G_ ratio of the CNTs increases after different ball-milling times, reflecting the increase in defects and damage to the integrity of the CNTs. The longer the ball-milling time, the deeper the damage to the integrity of the CNTs. The G-peaks of the CNTs ball-milled at different times are shifted to the right, which is due to the deformation of the structure caused by the thermal compression of CNTs during ball milling, and the length of the C=C bond is also reduced, so the peak is shifted to the right. To obtain composites with excellent mechanical properties, the ball-milling time should be controlled within an appropriate range to reduce damage to the CNTs.

#### 3.1.3. Analysis of Phase Composition of CNT/AlSi10Mg Composite Powder at Different Ball-Milling Times

**Figure 6:**
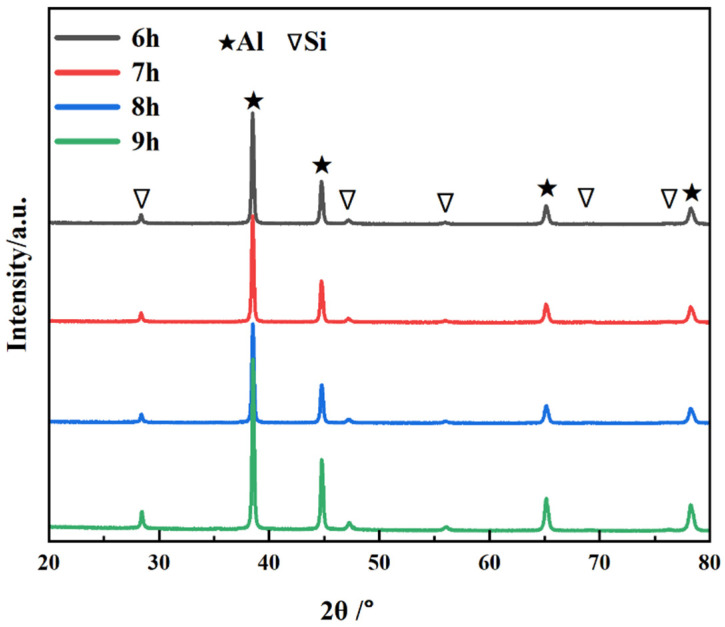
XRD patterns of CNT/AlSi10Mg composite powder with different ball-milling times.

Figure 6 shows the XRD patterns of the CNT/AlSi10Mg composite powder with different ball-milling times. The results indicate that with the extension of the ball-milling time from 6 to 9 h, most of the phases in the CNT/AlSi0Mg composite powder are Al, and a small amount of Si is contained in the AlSi10Mg powder. In addition, the diffraction peak corresponding to CNTs is not found on the atlas. This is because the amount of CNTs added is too small, and it is also mentioned above that CNTs are coated by Al particles after a long time of mechanical ball milling, so it is difficult to detect them. The diffraction peaks are pure Al and pure Si, indicating that no other impurities are introduced during the 6 h to 9 h mechanical ball-milling process.

#### 3.1.4. Mechanical Properties of CNT/AlSi10Mg Composite Powder at Different Ball-Milling Times

**Figure 7:**
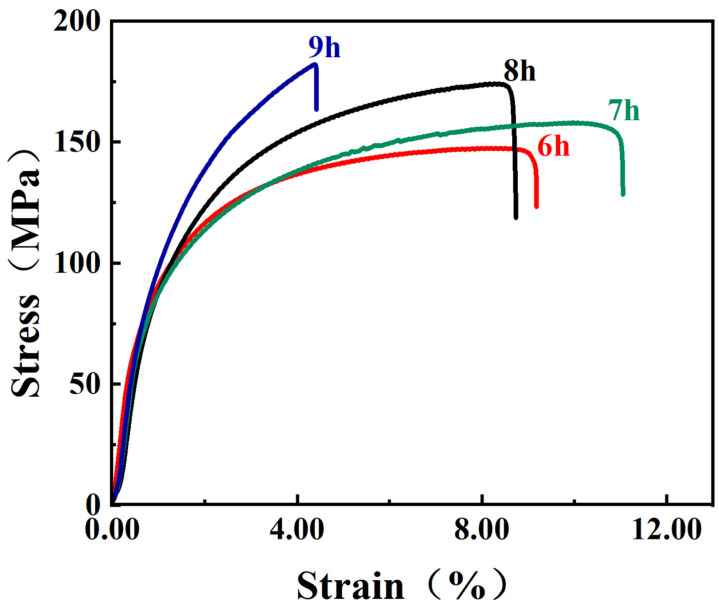
The stress–strain curves of CNT/AlSi10Mg composites with different ball-milling times.

**Figure 8:**
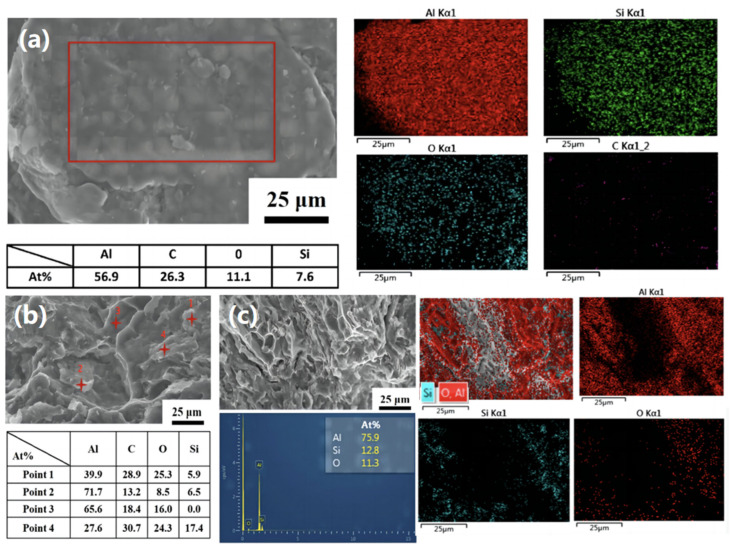
EDS surface energy spectrum of the composite material with 8 h and 9 h ball milling: (**a**) EDS point scan of the composite powder with 9 h ball milling. (**b**) EDS point scan of the tensile fracture with 9 h ball milling. (**c**) EDS face scan of the tensile fracture with 8 h ball milling.

Figure 7 shows the stress–strain curves of the CNT/AlSi10Mg composites at different ball-milling times. The results indicate that the strength of the composites increases with increasing ball-milling time. Among them, the tensile properties of the material prepared by ball milling of the powder for 8 h is the best, while the elongation of ball milling for 9 h is the lowest. Combined with Figure 3, it can be seen that the surface of the powder of ball milling for 9 h is oxidized to generate Al_2_O_3_, which reduces the elongation of the matrix to 4.2% after 9 h. Figure 8 shows the EDS spectra of the CNT/AlSi10Mg composite powder with tensile fracture after 9 h of ball milling, which confirms this result. Figure 8a shows that after 9 h of ball milling, a large amount of oxygen is introduced to the powder surface, which is discussed in the previous section. To prevent the powder from cold welding, anhydrous ethanol is added as a process control agent, so 9 h after the ball milling, oxygen is introduced to combine with Al to generate Al_2_O_3_, which reduces the elongation of the composite material to a minimum. Figure 8b shows the EDS spectra of the tensile fracture of the composites after 9 h of ball milling. Compared with the EDS spectrum of the 8 h tensile fracture of the ball mill, the oxygen content of the ball-milling process for 9 h is more, which shows that if the ball-milling time is too long, this introduces Al_2_O_3_ and reduces the performance of the material.

**Figure 9:**
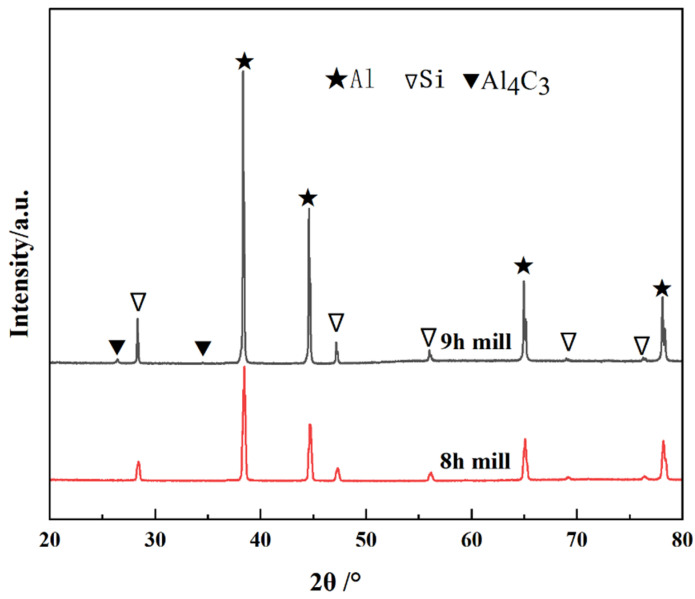
XRD patterns of CNT/AlSi10Mg composites with different ball-milling times.

**Figure 10:**
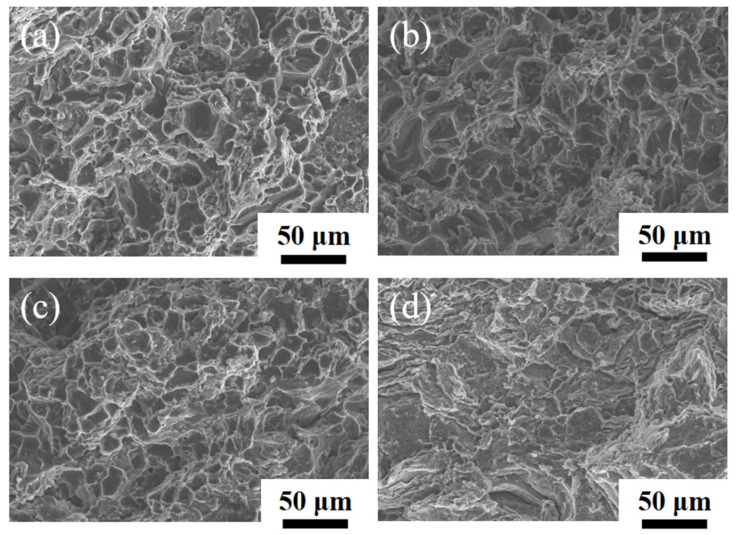
The fracture surfaces of CNT/AlSi10Mg composites with different ball-milling times: (**a**) 6 h. (**b**) 7 h. (**c**) 8 h. (**d**) 9 h.

Figure 9 shows XRD results of the CNT/AlSi10Mg composites at ball-milling times of 8 h and 9 h. It indicates that the intensity of the Al_4_C_3_ phase increases as the ball-milling time increases from 8 h to 9 h. This indicates that a small number of the CNTs react with the Al matrix to form brittle phase Al_4_C_3_ [17]. Kuzumaki et al. [15] reported that the CNTs were stable in the Al matrix and did not form carbide as long as they were high-quality and low defect tubes. However, ball-milling causes damage to the CNTs, thereby reducing the stability of the CNTs. Figure 10 shows the tensile fractures of the CNT/AlSi10Mg composites at different ball-milling times, with Figure 10a–c showing the fracture morphology after 6, 7, and 8 h of ball milling, respectively. It can be seen that the fracture surface is characterized by small dimples of uniform size and large tear ridges, showing obvious ductile fracture characteristics with further extension of the ball-milling time to 9 h. No evident tough nests are found (Figure 10d), and Al_2_O_3_ reduces the ductility of the material, resulting in a brittle fracture.

### 3.2. Effect of Different Carbon Nanotube Content on the Mechanical Properties of the Composites

#### 3.2.1. Analysis of SAED TEM Images Corresponding to 1.1 wt.% CNT/AlSi10Mg Composites

**Figure 11:**
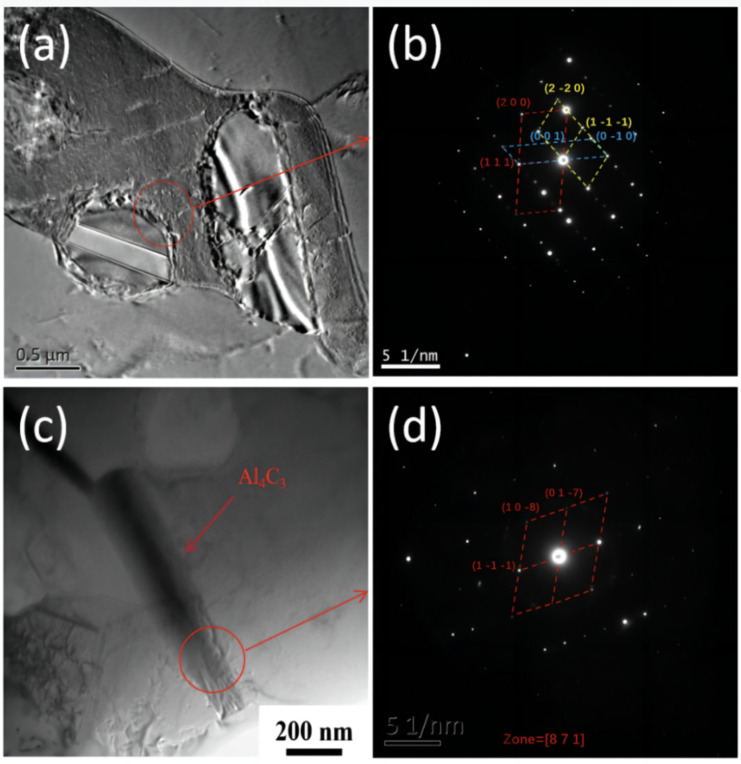
TEM images with the corresponding SAED of 1.1 wt.% CNT/AlSi10Mg composites (**a**) a bright TEM image of 1.1 wt.% CNT/AlSi10Mg composites. (**b**) SAED of corresponding image. (**c**) The image of Al_4_C_3_. (**d**) SAED of Al_4_C_3_.

Figure 11 shows the SAED TEM images corresponding to the 1.1 wt.% CNT/AlSi10Mg composites. As shown in Figure 11b, the selected area electron diffraction (SAED) pattern consists of diffraction spots of three single crystals: Al, Si, and Mg. These spots correspond to the (1 1 1) (2 0 0) crystallographic plane of Al, the (1 −1 −1) (2 −2 0) crystallographic plane of Si, and the (0 0 1) (0 −1 0) crystallographic plane of Mg, respectively, revealing the presence of the main metal of the composite. In the TEM of the 1.1 wt.% CNT/AlSi0Mg composite, we find the presence of Al_4_C_3_ (as in Figure 11c), which may be due to the interfacial reaction with the aluminum matrix caused by the addition of a higher amount of CNTs [4].

#### 3.2.2. Mechanical Properties of CNT/AlSi10Mg Composites with Different CNT Content

**Figure 12:**
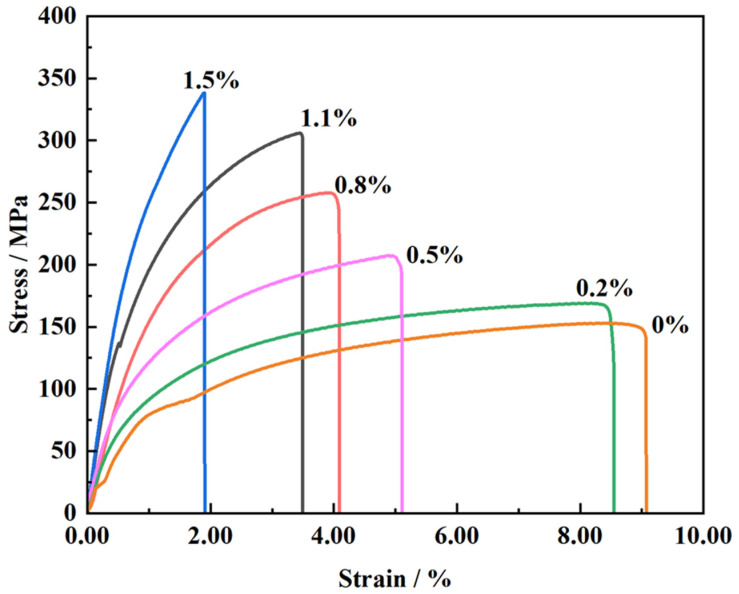
The stress–strain curves of CNT/AlSi10Mg composites with different CNT content.

Figure 12 depicts the stress–strain curves of the composites with varying CNT content. The results indicate that the mechanical properties of the composites are significantly affected by the amount of carbon nanotubes added. As the CNT content increases, the tensile strength of the composites initially increases, reaching a peak value at 1.5 wt.% CNT content (337 ± 5.2 MPa), which is 123% higher than that of the original matrix material (151 ± 2.1 MPa). This increase is attributed to the process hardening during ball milling, but, more importantly, due to the addition of a high content of carbon nanotubes. When the material is stressed, the carbon nanotubes can, on the one hand, bear part of the load for the matrix, and the high-energy ball milling causes the carbon nanotubes to encapsulate inside the aluminum matrix, and the CNTs entering inside the matrix play a bridging role between the aluminum grains, transferring the load through the bridging mechanism [28]. The plastic deformation and fracture processes of the aluminum matrix are inhibited, thus, improving the tensile strength of the composite. On the other hand, CNTs generally accumulate at grain boundaries, blocking them and refining the grains, which leads to higher-density and homogeneously dispersed dislocations, further improving the strength of the composites [29,30].

**Figure 13:**
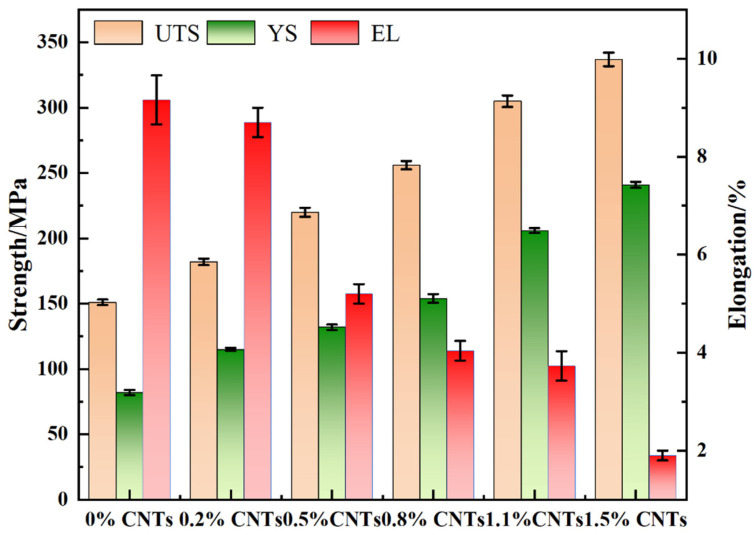
UTS, YS, and elongation plots of CNT/AlSi10Mg composites with different CNT content.

Table 1 and Figure 13 show the results of comparing the mechanical properties of different carbon nanotube composites. The results show that with the increase in carbon nanotube content, the tensile strength and yield strength of the composites are increased compared with the original matrix material. When the carbon nanotube content is 1.5 wt.%, the yield strength is up to 241 ± 2.3 MPa, which is 65% higher than that of the original material. However, when the CNT content exceeds 1.1 wt.%, the elongation of the composites starts to decrease rapidly. This is due to the excessive content of added carbon nanotubes, which may produce an agglomeration in the matrix and make the carbon nanotubes poorly bonded to the matrix interface, meaning it is difficult for them to function. On the other hand, during the sintering process, the CNTs react with the aluminum matrix to form the brittle phase Al_4_C_3_, as confirmed in Figure 9. The deformation ability of carbon nanotubes and the matrix material is not consistent, and the plastic deformation of the aluminum matrix is more strongly hindered by CNTs as the CNT content increases [31,32]. This is one reason why that the elongation of CNT/AlSi10Mg composites with high CNT content decreases significantly.

**Figure 14:**
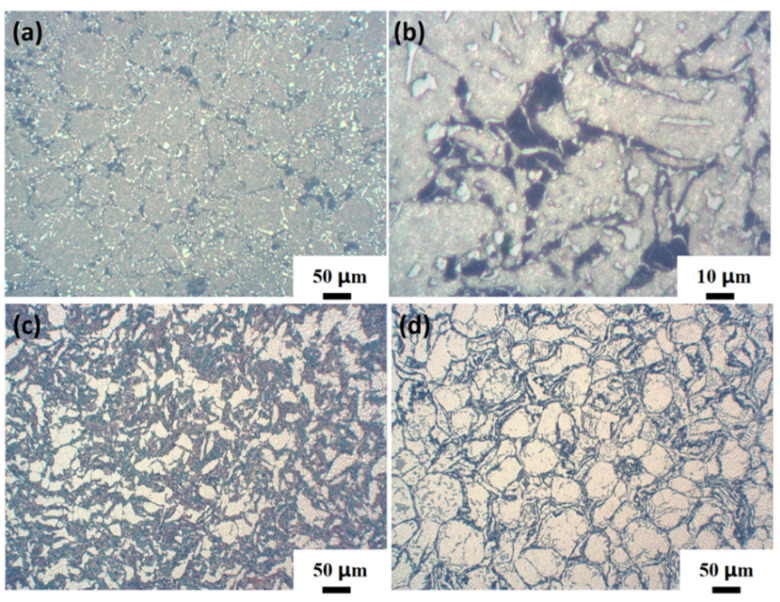
Metallographic structure of composite materials (**a**) original matrix AlSi10Mg. (**b**) An example of α-Al dendrite and coarse eutectic Si. (**c**) 0.2 wt.% CNTs. (**d**) 0.8 wt.% CNTs.

Figure 14 shows the metallographic structure of the composite with different CNT content. Figure 14a is the topography of the original non-ball-milled AlSi10Mg, and Figure 14b is the topography of the enlarged original material. It can be seen that the structure size of the original matrix material is uniform, and the surface is mainly distributed with fine α-Al dendrite and coarse eutectic Si. Figure 14c,d are metallographic diagrams of 0.2 wt.% CNT and 0.8 wt.% CNT/AlSi10Mg composites, respectively, from which it can be found that compared with the original matrix structure without ball milling, the grain of the composite after ball milling becomes larger and more eutectic Si is precipitated. Compared with the metallographic structure of 0.2 wt.% CNT composites, the grains of 0.8 wt.% CNT composites are more uniform, and there is no obvious coarse eutectic Si at the grain boundary. From the microstructure of 0.2 wt.% CNT composites, it can be seen that eutectic Si is agglomerated together and distributed on the Al phase in the form of coarse needles, while the eutectic Si of 0.8 wt.% CNT composites exists as fine fibers and is evenly distributed. This shows that the addition of CNTs can play the role of refining grains, and can effectively inhibit the precipitation and agglomeration of eutectic Si. This is because CNTs can act as a nucleation matrix in composite materials, increasing nucleation rate and reducing grain size. Secondly, CNTs can hinder the movement of grain boundaries, inhibit the growth of grains, and reduce grain size. This is also the reason why the tensile strength of 0.8 wt.% CNT/AlSi10Mg composites increases and the elongation decreases less.

**Figure 15:**
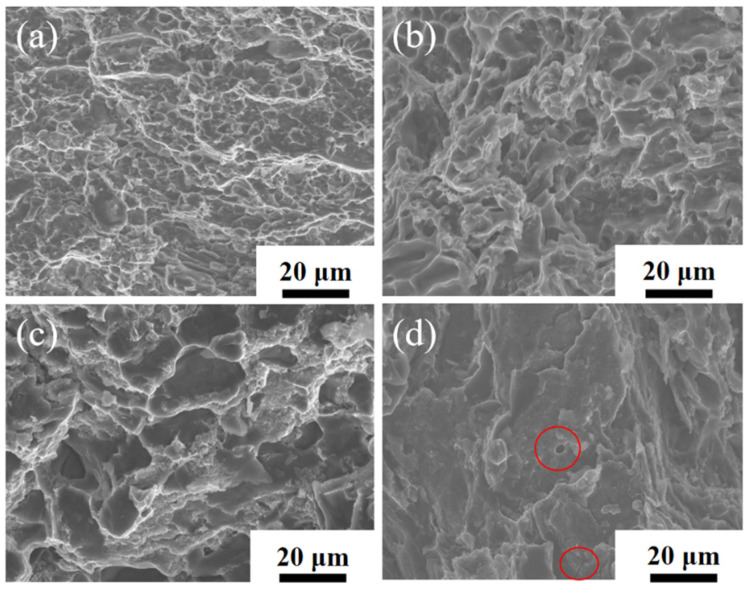
The fracture surfaces of CNT/AlSi10Mg composites with different CNT content: (**a**) 0.5 wt.% CNTs. (**b**) 0.8 wt.% CNTs. (**c**) 1.1 wt.% CNTs. (**d**) 1.5 wt.% CNTs.

Figure 15 shows the SEM images of the tensile fracture of the CNT/AlSi10Mg composites with different CNT content. Figure 15a–c show the fracture morphologies of 0.5 wt.% CNT, 0.8 wt.% CNT, and 1.0 wt.% CNT composites, respectively, and the fracture consists of a large number of tough nests, showing ductile fracture characteristics. Figure 15d shows the fracture morphology of 1.5 wt.% CNTs, which is composed of a few tough nests and most of the tearing folds, indicating brittle fracture. In addition to a small number of dimples, there are also some cracks and some very large pores in the fracture. The formation of these pores is caused by the aggregation in some areas due to high CNT content in the composite material. In the process of tensile fracture, the fracture preferentially occurs along these agglomeration areas, which results in several aluminum particles being pulled out as a whole, thus, forming large holes, which is another reason for the reduction in the elongation of high-content CNT composites.

### 3.3. Effect of Different Carbon Nanotube Content on the Corrosion Resistance of CNT/AlSi10Mg Composites

**Figure 16:**
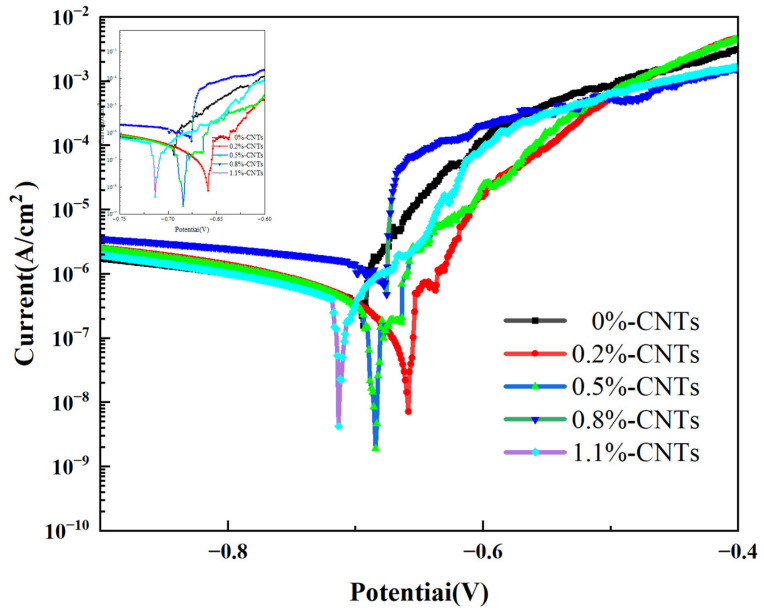
Anodic polarization curves of CNT/AlSi10Mg composites with different CNT content in 3.5 wt.% NaCl solution.

Figure 16 displays the anodic polarization curves of the CNT/AlSi10Mg composites with varying CNT content in a 3.5 wt.% NaCl solution. The overlapping curves are enlarged in the upper left corner of Figure 16 for better visualization. Table 2 presents the corresponding fitting results. The dynamic potential polarization curves of the five samples in Figure 16 exhibit similar shapes, indicating comparable electrochemical corrosion processes in the 3.5 wt.% NaCl solution. The polarization curves do not show passive regions; therefore, the type of corrosion of both materials in the 3.5 wt.% NaCl solution is an active corrosion process [33]. As shown in Figure 4 and Table 2, the composite materials with 0.2% and 0.5% CNT content exhibit slightly higher E_corr_ (corrosion potential) values compared to the original material without CNTs, while I_corr_ (corrosion current density) displays a decreasing trend. According to Faraday’s second law [34], there is a one-to-one correspondence between the corrosion current density and corrosion rate. A higher I_corr_ indicates a higher corrosion rate. However, the polarization curve fitting results show that the corrosion potential of the composite specimen is low, and the corrosion current density is not much different from that of the matrix specimen without CNTs. Therefore, the corrosion rate cannot be calculated.

**Figure 17:**
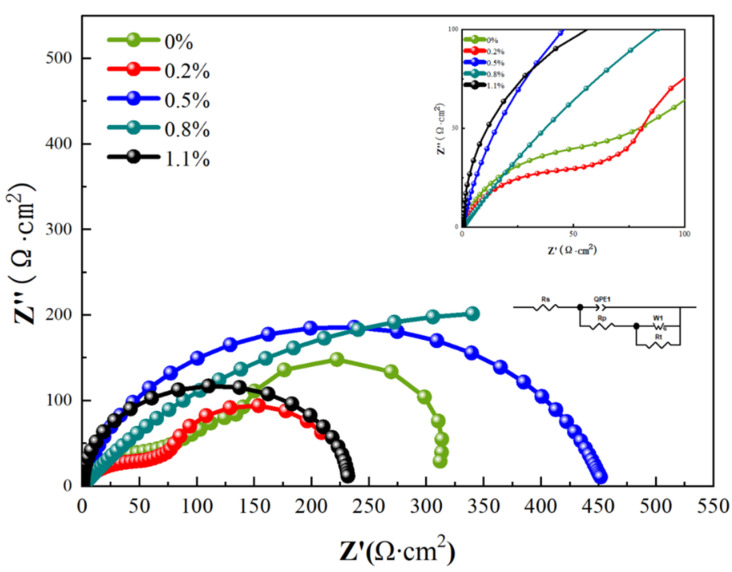
Electrochemical impedance spectra of CNT/AlSi10Mg composites with different CNT content in 3.5 wt.% NaCl solution.

Figure 17 shows the electrochemical impedance spectra (EIS) of CNT/AlSi10Mg composites with different CNT content, which show that the Nyquist plots of the 0 wt.% CNT/AlSi10Mg and 0.2 wt.% CNT/AlSi10Mg composites consist of two distinct capacitance rings. The 0.8 wt.% CNT/AlSi10Mg composite has the largest arc radius and the 0.2 wt.% CNT/AlSi10Mg composite has the smallest arc radius. A larger diameter of the capacitive reactance indicates a higher impedance of the oxide film. The inductive loops of all the samples are affected by Cl^−^ adsorption caused by the destruction of the oxide film on the surface of the samples [35]. Therefore, the 0.8 wt.% CNT/AlSi10Mg composite has the largest radius of circle, largest polarization resistance, and highest corrosion resistance, while the 0.2 wt.% CNT/AlSi10Mg composite has the smallest radius of circle, smallest polarization resistance, and worst corrosion resistance. The electrochemical impedance spectra were analyzed and fitted using ZSimpWin software, and the equivalent circuit fitting results are shown in Figure 17.

**Figure 18:**
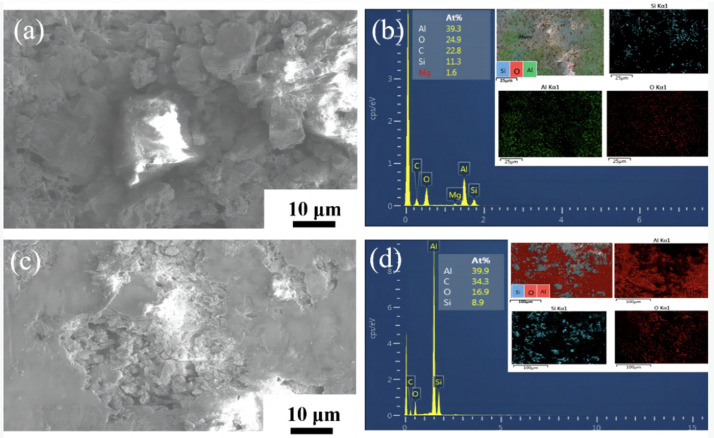
SEM images of composites reinforced with different CNT ratios after the corrosion test in 3.5 wt.% NaCl: (**a**) 0.2 wt.% CNT/AlSi10M corrosion profile. (**b**) 0.2 wt.% CNT/AlSi10M EDS surface scan of corrosion profile. (**c**) 0.8 wt.% CNT/AlSi10M corrosion profile. (**d**) 0.8 wt.% CNT/AlSi10Mg EDS surface scan of corrosion profile.

Figure 18 shows the corrosion morphology of CNT/AlSi10Mg composites with different CNT content. Figure 18a shows the corrosion morphology of the 0.2 wt.% CNT/AlSi10Mg composites. After the 0.2 wt.% CNT/AlSi10Mg composite specimens are corroded by the 3.5 wt.% NaCl solution, the surface oxide layer film is destroyed and the internal Si particles are exposed. This is because when a small amount of CNT is added, a potential difference is formed between the CNTs and Al matrix [36], which forms an electrochemical cell with the aluminum matrix and causes galvanic corrosion. Additionally, the presence of the second phase increases the pores at the composite interface, making it easier for chloride ions to penetrate and reducing the material’s corrosion resistance [37]. Figure 18b reveals that the corrosion products are primarily Al_2_O_3_. Figure 18c shows the corrosion morphology of the 0.8 wt.% CNT/AlSi10Mg, which exhibits pitting corrosion. Compared to the 0.2 wt.% CNT/AlSi10Mg composite corrosion morphology, the 0.8 wt.% CNT/AlSi10Mg composite corrosion pits are smaller and the corrosion rate is lower, which is due to the addition of the appropriate amount of CNTs that are uniformly dispersed in the Al matrix. The good corrosion resistance of CNTs can further hinder the corrosion and improve the corrosion resistance of the material, which is consistent with the conclusions obtained in Figure 16 and Figure 17.

## 4. Conclusions

The present study demonstrates the preparation of CNT/AlSi10Mg composites using a high-energy ball-milling method combined with SPS, and the following conclusions can be drawn from the study of their properties.

After continuous extrusion and shearing, the aluminum powder turns into flakes and the surface area increases continuously, which provides a large number of attachment sites for the CNTs. Moreover, the mechanical force generated during the ball-milling process cuts off and disperses CNTs, leading to improved dispersion of CNTs in the aluminum matrix and enhanced mechanical properties of the composites. However, excessive ball-milling time damages the CNT structure, resulting in unsatisfactory reinforcement. Therefore, the optimal ball-milling time is determined to be 8 h.

The tensile strength of the CNT/AlSi10Mg composite increases with increasing CNT content. The composite exhibits a maximum tensile strength of 337 ± 5.2 MPa and yield strength of 241 ± 2.3 MPa at CNT mass fractions of 1.5 wt.%.

The addition of 0.2 wt.% CNTs results in galvanic coupling corrosion due to the small amount of CNTs, which reduces the corrosion resistance of the composites. However, the uniform dispersion of 0.8 wt.% CNTs on the surface of the matrix hinders corrosion and improves the mechanical properties of the composites. The addition of CNTs can effectively inhibit the precipitation of Si and improve the performance of the composite.

## Figures and Tables

**Table 1:** Comparison of various mechanical properties of composites with different CNT content.

Alloy	UTS (MPa)	YS (MPa)	EL (%)
0%	151 ± 2.1	82 ± 2.0	9.16 ± 0.5
0.2%	182 ± 2.5	115 ± 1.2	8.7 ± 0.3
0.5%	220 ± 3.4	132 ± 2.1	5.2 ± 0.2
0.8%	256 ± 3.2	154 ± 3.4	4.04 ± 0.2
1.1%	305 ± 4.4	206 ± 1.8	3.73 ± 0.3
1.5%	337 ± 5.2	241 ± 2.3	1.9 ± 0.1

**Table 2:** Fitting results of polarization curves for composites with different CNT content.

Alloy	0 wt.%	0.2 wt.%	0.5 wt.%	0.8 wt.%	1.1 wt.%
E_corr_ (V)	−0.695	−0.654	−0.686	−0.677	−0.719
I_corr_ (A/cm^2^)	−6.312	−6.301	−6.361	−6.346	−6.322
